# Supplementary material for: Learning curve and surgical outcome of robotic assisted colorectal surgery with ERAS program
Source: Sci Rep. 2022 Nov 29;12:20566. doi: 10.1038/s41598-022-24665-w (PMC9709162; doi:10.1038/s41598-022-24665-w)
Supplement: Supplementary file 2 — Supplementary Information 2. [file 41598_2022_24665_MOESM2_ESM.zip › RAL Raw data-Ñ[▒K/087/HS_ñw╝╨░O▒Kñσ.pdf]

正式摘要(電子簽章：000000218247027)

W52 / W52 ☐ 英文標題(注意:無英文姓名-請補病人基本檔!) ☐ 【讀取】憑證

臺中榮民總醫院

出院病歷摘要

轉入號： 轉出號：

[redacted]

(8)流水編號	
(9)入院日期	110/05/28 (2021)
(10)轉科日期	
(11)出院日期	110/06/01 (2021) 住院天數計 4 日

診斷

- 入院 :1. R/O S-colon Ca, 30cm AAV, s/p polypectomy at LMD, tubovillous adenoma with malignant change
  2. Colon polyps
- 出院 :Colon polyps R/O S-colon Ca, 30cm AAV, s/p polypectomy at LMD, tubovillous adenoma with malignant change s/p roboric assisted anterior resection on 2021/05/29

主訴

- FOB+ in 2021/01

病史

- This 60 y/o male with a hsitory of HTN and DM under medications accidentally found occult blood test positive in health check in 2021/01. He then went to CRS OPD for help where colonscopy showed =1 sessile polyp at 60cm AAV, s/p EMR + hemiclipping*2, previous polypectomy wound at 30cm AAV, s/p biopsy + tatto + hemoclip localization, 1 polyp at 15cm AAV, s/p EMR. The pathology shows tubular adenoma. However, some abortive glands with inflammation is worrisome for invasive carcinoma. As a result, he was admitted for robotic assisted anterior resection.

體檢發現

-
  - General appearance :
    - easy looking , conscious clear , without cardiopulmonary distress
    - Vital signs自動帶入date 體溫(BT) 脈搏(P) 呼吸(R) 血壓(BP) 身高(BH) 體重(BW) BMI
      20210528 36.2C 90/min 20/min 142/87mmHg 175.5cm 70.4kg 22.9
    - Vital signs: B.P. 142/87 mmHg , P.R. 90 /min , R.R. 20 /min , B.T. 36.2 ℃
  - HEENT :
    - Eyes: no anemic conjunctiva, no icteric sclera, normal EOM
    - ENT: no otorrhea, no rhinorrhea, no erythematous mucosa, no injected tonsil, no deformity
  - Neck: supple, freely movable, trachea midline,no deformity, no engorged jugular vein , no palpable thyroid or lymph node enlargement
  - Chest:
    - Inspection: Grossly normal, symmetric and fully expansion, no accessory muscle used, no operation scar, no spider angiomata, no gynecomastia
    - Palpation: no palpable mass or nodule, no subcutaneous emphysema, no tenderness
    - Ausculation: clear breathing sound
  - Heart:
    - Percussion: normal heart size
    - Ausculation: regular heart beat, normal S1, S2, no gallop, no murmur
  - Abdomen:
    - Inspection: in
      - flat contour, symetrically, no scar, no stria, no dilated veins, no rashes
    - Ausculation: normoactive bowel sound, no abdominal bruits
    - palpation: soft, no muscle guarding or rigidity, no local tenderness, no rebounding pain, no palpable mass, no palpable pulsation
    - Percussion: no shifting dulllness, no hepatomegaly, no splenomagaly

-
  -

Peripheral pulsation :	CA	BA	RA	FA	PA	PTA	DPA
Right side :	++	++	++	++	++	++	++/+++
Left side :	++	++	++	++	++	++	++/+++

  - Genito-anus: no tenderness or palpable mass lesion or rectal shelf, normal external genitalia, normal anal tone, brown stool

CRS CONDITION

-
  - Lung

手術日期及方法

- 手術日期及方法20210529 Robot Assisted Colectomy(L)

住院治療經過

- After admission, pre op survey was ok and operation was done smoothly. He tolerated the procedure well and could stand oral feeding gradually. Under relativelty stable conditions, he will be discharged.

合併症

一般檢查

- 累積報告 :

DATE	TIME	ALB	TP	BIL.T	BIL.D	ALKP	AST	ALT	LDH	AMY	LIPASE	NH3	Ferritin
1100529	1404	4.0		0.76	0.21	64	15	17					

DATE	TIME	WBC	RBC	HGB	HCT	MCV	PLT	BLST	NEUT	BAND	SEG	LYM	MON	EOS	BAS
1100529	1404	15340	4.66	13.6	40.3	86.5	224		92.9			4.9	0.9	1.2	0.1

DATE	TIME	NA	K	CL	BUN	CREAT	eGFR	CA	Mg	P	CRP	ESR	PCT	D-Dimer	LACTATE
1100529	1404	138	3.9	104	8	0.81	103.31	9.0							

DATE	TIME	GLUCOSE	HbA1C	CHOL	TG	HDL	LDL	KETONE	CK	CKMB	TRO-T	PT-P	APTT-P	NT-pro BNP
1100529	1404	202												

特殊檢查

病理報告

其它

出院時情況

- 3.改門診治療

出院指示

-

藥名	劑量	劑量單位	用法	頻次	數量	數量單位	現況	天數	單位
(口)Takepron OD tab 30mg	1	TAB	PO	QDAC	7	TAB	TH	7	天
Mag. Oxide tab	2	TAB	PO	TID	42	TAB	TH	7	天
Focus geL 40gm		TUB	TOP	TID	0	TUB	TH	1	瓶
5/160mg Exforge tab	1	TAB	PO	QD	7	TAB	TH	7	天
Repaglinide tab 1mg	1	TAB	PO	BID	14	TAB	TH	7	天
Acetal tab 500mg	1	TAB	PO	Q6H	28	TAB	TH	7	天

出院計劃

主治醫師 2062F 蔣鋒帆 / 住院醫師 2309E 古朋育

正式摘要(電子簽章：000000218247027)

W52 / W52

doctorRank:B
